# Supplementary material for: The molecular basis of extensively drug-resistant Salmonella Typhi isolates from pediatric septicemia patients
Source: PLoS One. 2021 Sep 28;16(9):e0257744. doi: 10.1371/journal.pone.0257744 (PMC8478237; doi:10.1371/journal.pone.0257744)
Supplement: S1 Table — (DOCX) [file pone.0257744.s002.docx]

**S1 Table. The minimum inhibitory concentration (MIC) results (µg/ml) of all samples used in this study, related to Fig 1.**

| # | Age* | Sex | Wards  ** | AMP*** | SXT | CIP | CTX | CRO | PIP/TZB | AMC | AZM | IPM | MEM |
| --- | --- | --- | --- | --- | --- | --- | --- | --- | --- | --- | --- | --- | --- |
| 1 | 5 | M | E | ≥32 | ≥4/76 | ≥64 | 8 | 32 | 32/4 - 64/4 | ≥32/16 | 8 | 0.25 | 0.25 |
| 2 | 2 | M | I | ≥32 | ≥4/76 | 32 | 8 | ≥64 | 4/4-128/4 | 8/4 | 2 | 0.5 | 0.25 |
| 3 | 4 | F | E | ≥32 | ≥4/76 | ≥64 | ≥64 | 32 | 32/4 - 64/4 | 16/8 | 8 | 0.5 | 0.25 |
| 4 | 10 | M | I | ≥32 | ≥4/76 | 32 | 32 | 8 | 32/4 - 64/4 | 16/8 | 4 | 0.5 | 0.25 |
| 5 | 11 | M | M | ≥32 | ≥4/76 | 8 | ≥64 | ≥64 | 32/4 - 64/4 | ≥32/16 | 4 | 0.25 | 0.5 |
| 6 | 11 | M | C | ≥32 | ≥4/76 | ≥64 | 32 | ≥64 | 4/4-128/4 | 8/4 | 4 | 0.25 | 0.5 |
| 7 | 13 | M | E | ≥32 | ≥4/76 | ≥64 | ≥64 | 32 | 4/4-128/4 | 4/2 | 4 | 0.25 | 0.5 |
| 8 | 11 | M | E | ≥32 | ≥4/76 | 32 | ≥64 | ≥64 | ≥128/4 | ≥32/16 | 4 | 0.5 | 0.5 |
| 9 | 3 | M | C | ≥32 | ≥4/76 | 8 | ≥64 | ≥64 | 4/4-128/4 | 4/2 | 4 | 0.25 | 0.5 |
| 10 | 4 | M | E | ≥32 | ≥4/76 | ≥64 | 32 | ≥64 | ≥128/4 | ≥32/16 | 4 | 0.5 | 0.5 |
| 11 | 4 | F | G | ≥32 | ≥4/76 | ≥64 | 32 | 32 | ≥128/4 | ≥32/16 | 2 | 0.5 | 0.5 |
| 12 | 4 | F | E | ≥32 | ≥4/76 | ≥64 | 8 | ≥64 | 32/4 - 64/4 | 16/8 | 2 | 0.5 | 0.25 |
| 13 | 5 | M | O | ≥32 | ≥4/76 | 32 | ≥64 | 32 | ≥128/4 | 16/8 | 2 | 0.5 | 0.5 |
| 14 | 4 | M | I | ≥32 | ≥4/76 | ≥64 | ≥64 | ≥64 | ≥128/4 | 16/8 | 8 | 0.5 | 0.5 |
| 15 | 9 | F | O | ≥32 | ≥4/76 | 32 | 32 | ≥64 | 4/4-128/4 | 8/4 | 2 | 0.5 | 0.5 |
| 16 | 2 | F | O | ≥32 | ≥4/76 | ≥64 | ≥64 | ≥64 | ≥128/4 | 16/8 | 8 | 0.5 | 0.25 |
| 17 | 3 | M | E | ≥32 | ≥4/76 | ≥64 | ≥64 | 32 | 32/4 - 64/4 | ≥32/16 | 2 | 0.25 | 0.25 |
| 18 | 6 | M | C | ≥32 | ≥4/76 | ≥64 | ≥64 | 32 | 32/4 - 64/4 | 16/8 | 8 | 0.25 | 0.25 |
| 19 | 8 | M | C | ≥32 | ≥4/76 | ≥64 | ≥64 | ≥64 | ≥128/4 | 16/8 | 8 | 0.25 | 0.25 |
| 20 | 2 | M | W | ≥32 | ≥4/76 | 32 | ≥64 | 32 | 32/4 - 64/4 | 16/8 | 8 | 0.25 | 0.25 |
| 21 | 6 | M | W | ≥32 | ≥4/76 | ≥64 | 32 | ≥64 | ≥128/4 | ≥32/16 | 2 | 0.25 | 0.25 |
| 22 | 5 | M | E | ≥32 | ≥4/76 | ≥64 | ≥64 | 32 | 32/4 - 64/4 | ≥32/16 | 8 | 0.5 | 0.25 |
| 23 | 2 | M | I | ≥32 | ≥4/76 | ≥64 | ≥64 | ≥64 | 32/4 - 64/4 | 16/8 | 4 | 0.5 | 0.5 |
| 24 | 7 | M | MI | ≥32 | ≥4/76 | ≥64 | 8 | 32 | 32/4 - 64/4 | ≥32/16 | 8 | 0.5 | 0.5 |
| 25 | 5 | M | E | ≥32 | ≥4/76 | 8 | ≥64 | ≥64 | ≥128/4 | 16/8 | 4 | 0.25 | 0.25 |
| 26 | 11 | M | E | ≥32 | ≥4/76 | ≥64 | ≥64 | 32 | ≥128/4 | 16/8 | 8 | 0.25 | 0.25 |
| 27 | 2 | M | E | ≥32 | ≥4/76 | ≥64 | ≥64 | 32 | ≥128/4 | ≥32/16 | 8 | 0.25 | 0.5 |
| 28 | 7 | M | W | ≥32 | ≥4/76 | ≥64 | 32 | ≥64 | ≥128/4 | 16/8 | 4 | 0.5 | 0.5 |
| 29 | 2 | F | W | ≥32 | ≥4/76 | ≥64 | 32 | 8 | 4/4-128/4 | 8/4 | 4 | 0.5 | 0.25 |
| 30 | 6 | M | E | ≥32 | ≥4/76 | ≥64 | ≥64 | ≥64 | 32/4 - 64/4 | 16/8 | 4 | 0.5 | 0.25 |
| 31 | 3 | M | C | ≥32 | ≥4/76 | ≥64 | ≥64 | 8 | 32/4 - 64/4 | ≥32/16 | 8 | 0.25 | 0.25 |
| 32 | 5 | M | E | ≥32 | ≥4/76 | ≥64 | 32 | 32 | 32/4 - 64/4 | 16/8 | 4 | 0.25 | 0.25 |
| 33 | 3 | F | E | ≥32 | ≥4/76 | ≥64 | ≥64 | ≥64 | 32/4 - 64/4 | 16/8 | 4 | 0.25 | 0.25 |
| 34 | 6 | M | E | ≥32 | ≥4/76 | 32 | ≥64 | ≥64 | 32/4 - 64/4 | ≥32/16 | 4 | 0.5 | 0.5 |
| 35 | 6 | F | O | ≥32 | ≥4/76 | ≥64 | ≥64 | ≥64 | 32/4 - 64/4 | 8/4 | 4 | 0.25 | 0.5 |
| 36 | 11 | M | C | ≥32 | ≥4/76 | ≥64 | ≥64 | 8 | ≥128/4 | 8/4 | 8 | 0.25 | 0.5 |
| 37 | 2 | F | W | ≥32 | ≥4/76 | 32 | 32 | ≥64 | 4/4-128/4 | 8/4 | 4 | 0.5 | 0.5 |
| 38 | 13 | F | F | ≥32 | ≥4/76 | 32 | ≥64 | ≥64 | 32/4 - 64/4 | 8/4 | 4 | 0.5 | 0.5 |
| 39 | 10 | F | E | ≥32 | ≥4/76 | ≥64 | ≥64 | 8 | 32/4 - 64/4 | 8/4 | 8 | 0.5 | 0.25 |
| 40 | 4 | F | O | ≥32 | ≥4/76 | 8 | ≥64 | ≥64 | 32/4 - 64/4 | 8/4 | 4 | 0.25 | 0.25 |
| 41 | 5 | M | O | ≥32 | ≥4/76 | ≥64 | ≥64 | 32 | ≥128/4 | ≥32/16 | 8 | 0.25 | 0.5 |
| 42 | 5 | M | E | ≥32 | ≥4/76 | ≥64 | 32 | ≥64 | 32/4 - 64/4 | 16/8 | 4 | 0.25 | 0.25 |
| 43 | 8 | F | E | ≥32 | ≥4/76 | 32 | 32 | ≥64 | ≥128/4 | 8/4 | 4 | 0.25 | 0.5 |
| 44 | 2 | F | O | ≥32 | ≥4/76 | ≥64 | ≥64 | ≥64 | 32/4 - 64/4 | ≥32/16 | 4 | 0.5 | 0.25 |
| 45 | 1 | M | E | ≥32 | ≥4/76 | ≥64 | 8 | 32 | 32/4 - 64/4 | ≥32/16 | 4 | 0.25 | 0.25 |

*, Age in years.

**, E, Peads Emergency; I, PAED-intensive care unit (ICU); M, Male Medical; C, Clinical Laboratory; G, General outpatient department (OPD); O, Peads Medical OPD; W, Pead Medical Ward; MI, Pead Medical ICU; F, Fatima Memorial Hospital (FMH)-executive clinic.

***, Antibiotic acronym: AMP, ampicillin, SXT, trimethoprim-sulfamethoxazole, CIP, ciprofloxacin, CTX, cefotaxime, CRO, ceftriaxone, AZM, azithromycin, PIP, piperacillin, TZB, tazobactam, AMC, amoxicillin/ clavulanic acid, IPM, imipenem, MEM, meropenem.
