# Supplementary material for: The molecular basis of extensively drug-resistant Salmonella Typhi isolates from pediatric septicemia patients
Source: PLoS One. 2021 Sep 28;16(9):e0257744. doi: 10.1371/journal.pone.0257744 (PMC8478237; doi:10.1371/journal.pone.0257744)
Supplement: S2 Table — (DOCX) [file pone.0257744.s003.docx]

**S2 Table. Antibiotic breakpoints, related to Fig 1 and S1 Table.**

| **Antibiotics** | **Antibiotic breakpoints** | | |
| --- | --- | --- | --- |
|  | **S** | **I** | **R** |
| **AMP** | ≤8 | 16 | ≥32 |
| **SXT** | ≤2/38 | - | ≥4/76 |
| **CIP** | ≤1 | 2 | ≥4 |
| **CTX** | ≤1 | 2 | ≥4 |
| **CRO** | ≤1 | 2 | ≥4 |
| **PIP/TZB** | ≤16/4 | 32/4-64/4 | ≥128/4 |
| **AMC** | ≤8/4 | 16/8 | ≥32/16 |
| **AZM** | ≤16 | - | ≥32 |
| **IPM** | ≤1 | 2 | ≥4 |
| **MEM** | ≤1 | 2 | ≥4 |

Antibiotic acronym: AMP, ampicillin, SXT, trimethoprim-sulfamethoxazole, CIP, ciprofloxacin, CTX, cefotaxime, CRO, ceftriaxone, AZM, azithromycin, PIP, piperacillin, TZB, tazobactam, AMC, amoxicillin/ clavulanic acid, IPM, imipenem, MEM, meropenem.
