# Supplementary material for: The molecular basis of extensively drug-resistant Salmonella Typhi isolates from pediatric septicemia patients
Source: PLoS One. 2021 Sep 28;16(9):e0257744. doi: 10.1371/journal.pone.0257744 (PMC8478237; doi:10.1371/journal.pone.0257744)
Supplement: S3 Table — (DOCX) [file pone.0257744.s004.docx]

**S3 Table. Select of samples for molecular characterization, related to Figs 2-6.**

| Lab No | Age (Yr) | Sex | AMP | SXT | CIP | CTX | CRO | PIP/TZB | AMC/CLA | AZM | IPM | MEM |
| --- | --- | --- | --- | --- | --- | --- | --- | --- | --- | --- | --- | --- |
|  |  |  | MDR | | XDR | | |  |  |  |  |  |
| Toddlers (1-2 yrs) | | | | | | | | | | | | |
| 16 | 2 | F | R | R | R | R | R | I | I | S | S | S |
| 2 | 2 | M | R | R | R | R | R | S | S | S | S | S |
| 20 | 2 | M | R | R | R | R | R | I | I | S | S | S |
| 23 | 2 | M | R | R | R | R | R | I | I | S | S | S |
| 27 | 2 | M | R | R | R | R | R | I | I | S | S | S |
| Pre-schoolers (3-5 yrs) | | | | | | | | | | | | |
| 33 | 3 | F | R | R | R | R | R | I | I | S | S | S |
| 9 | 3 | M | R | R | R | R | R | S | S | S | S | S |
| 17 | 3 | M | R | R | R | R | R | I | I | S | S | S |
| 31 | 3 | M | R | R | R | R | R | I | I | S | S | S |
| 40 | 4 | F | R | R | R | R | R | I | S | S | S | S |
| 12 | 4 | F | R | R | R | R | R | I | I | S | S | S |
| 1 | 5 | M | R | R | R | R | R | I | I | S | S | S |
| School-age children (6-12 yrs) | | | | | | | | | | | | |
| 15 | 9 | F | R | R | R | R | R | S | S | S | S | S |
| 6 | 11 | M | R | R | R | R | R | S | S | S | S | S |
| 8 | 11 | M | R | R | R | R | R | I | I | S | S | S |
| 36 | 11 | M | R | R | R | R | R | I | S | S | S | S |
| Adolescents (13-18 yrs) | | | | | | | | | | | | |
| 38 | 13 | F | R | R | R | R | R | I | S | S | S | S |
| 7 | 13 | M | R | R | R | R | R | S | S | S | S | S |
