# Supplementary material for: The molecular basis of extensively drug-resistant Salmonella Typhi isolates from pediatric septicemia patients
Source: PLoS One. 2021 Sep 28;16(9):e0257744. doi: 10.1371/journal.pone.0257744 (PMC8478237; doi:10.1371/journal.pone.0257744)
Supplement: S4 Table — (DOCX) [file pone.0257744.s005.docx]

**S4 Table. PCR and sequencing primers and PCR conditions used in this study, related to Methods.**

| Genes | Primers | Primer sequences (5’-3’) | Annealing temp. | Extension time (min) | Amplicon size (bp) |
| --- | --- | --- | --- | --- | --- |
| *pltB* | Forward | TAAACCATGATAGACTGG | 55℃ | 0.5 | 657 |
|  | Reverse | GAAAGTTACGGTTATACC |  |  |  |
|  | Sequencing | TAAACCATGATAGACTGG |  |  |  |
| *bla*_TEM1_ | Forward | AACCCTGGTAAATGCTTC | 55℃ | 1 | 930 |
|  | Reverse | GTATATATGAGTAAACTTGG |  |  |  |
| *catA1* | Forward | GAAGATCACTTCGCAGAATAA | 45℃ | 1 | 1003 |
|  | Reverse | CAGCAATAGACATAAGCG |  |  |  |
| *dhfR7* | Forward | GCAACGTCAGAAAATGGC | 60℃ | 0.5 | 405 |
|  | Reverse | AAACTGCTCAAAAAGGAAATTGA |  |  |  |
| *sul1* | Forward | GTATTGCGCCGCTCTTAGAC | 60℃ | 0.5 | 500 |
|  | Reverse | AGGGTTTCCGAGAAGGTGAT |  |  |  |
| *qnrS* | Forward | TATAATGGTAGTCTAGCCC | 52℃ | 1 | 722 |
|  | Reverse | GATGTGTGATTTTAAACG |  |  |  |
| *gyrA* | Forward | CTTTGAATCCGGGATACAG | 55℃ | 2 | 2726 |
|  | Reverse | TTCCATAGACAAGAAAAAGG |  |  |  |
|  | Sequencing | CTTTGAATCCGGGATACAG |  |  |  |
| *gyrB* | Forward | GAAAAGGGTAAAATAACGG | 55℃ | 2 | 2515 |
|  | Reverse | CATCATGATGCCCTGGCCAG |  |  |  |
|  | Sequencing | GAATAAAACGCCGATCCAC |  |  |  |
| *parC* | Forward | ATAGGGTATTATCTGCGGC | 55℃ | 2.5 | 2473 |
|  | Reverse | GAATAAACAACGGTTTTACG |  |  |  |
|  | Sequencing | ATAGGGTATTATCTGCGGC |  |  |  |
| *parE* | Forward | TGCACAGTTGCTGACAATC | 55℃ | 2 | 1990 |
|  | Reverse | TCGGATTCTCTTATCCGGCCTG |  |  |  |
|  | Sequencing | CTGTGGCTGAACCAGAAC |  |  |  |
| *bla*_CTX-M-15_ | Forward | GATGTGCAGCACCAGTAAAG | 52℃ | 0.5 | 549 |
|  | Reverse | AACGATATCGCGGTGATCTG |  |  |  |
| *macA* | Forward | CTGTAAGCTGTGTCATGATCG | 52℃ | 1 | 1308 |
|  | Reverse | CTCACATTGCACAGTTCAAGC |  |  |  |
|  | Sequencing | CTGTAAGCTGTGTCATGATCG |  |  |  |
| *acrB(N-term)* | Forward | GGTTAAAGTGCAGGAAATTACCG | 50℃ | 1.5 | 1695 |
|  | Reverse | CTACGCTATCGGTGTAGTGAT |  |  |  |
| *acrB(C-term)* | Forward | GACGATGCTCAAACCCGT | 50℃ | 1.5 | 1815 |
|  | Reverse | GCCAACTTTCCTAAGAAAAAGCC |  |  |  |
|  | Sequencing | GACTTCGAGTTGATTGACCA |  |  |  |
| *acrR* | Forward | CACCGACATATGGCACGAA | 52℃ | 1 | 633 |
|  | Reverse | CAGCGTCGGACACAATTGATA |  |  |  |
|  | Sequencing | GAAAGTTACGATCGGATTGA |  |  |  |

Associated gene functions are indicated here, as well as in the Result section when the corresponding results are discussed. *pltB*, the glycan-receptor binding subunit of typhoid toxin. *bla*_TEM1_, β-lactamase associated with resistance to β-lactam antibiotics. *catA1*, chloramphenicol acetyltransferase associated with chloramphenicol resistance. *dhfR7*, dihydrofolate synthetase associated with resistance to trimethoprim. *sul1*, dihydropteroate synthase associated with resistance to sulfamethoxazole. *qnrS*, quinolone resistance. *gyrA*, topoisomerase II. Some point mutations are associated with quinolone resistance. *gyrB*, topoisomerase II. Some point mutations are associated with quinolone resistance. *parC*, topoisomerase IV. Some point mutations are associated with quinolone resistance. *parE*, topoisomerase IV. Some point mutations are associated with quinolone resistance. *bla*_CTX-M-15_, β-lactamase associated with resistance to β-lactam antibiotics; *macA*, ABC-type efflux pump component. *acrB(N-term)* & *acrB(C-term)*, RND-type efflux pump component. One gene is artificially split into two pieces for efficient PCR amplification. *acrR*, a repressor for the *acrAB* operon (RND-type efflux pump component).
