# Supplementary material for: The molecular basis of extensively drug-resistant Salmonella Typhi isolates from pediatric septicemia patients
Source: PLoS One. 2021 Sep 28;16(9):e0257744. doi: 10.1371/journal.pone.0257744 (PMC8478237; doi:10.1371/journal.pone.0257744)
Supplement: S5 Table — (DOCX) [file pone.0257744.s006.docx]

**S5 Table. Details of the 107 completed *S.* Typhi genomes used in the study, related to Fig 6.**

| **Accession number** | **Assembly number** | **BioProject** | **BioSample** | **BioSample ID** | **Serovar** |
| --- | --- | --- | --- | --- | --- |
| GCF_000007545.1 | ASM754v1 | PRJNA371 | SAMN02604095 | 2604095 | Typhi |
| GCF_000195995.1 | ASM19599v1 | PRJNA236 | SAMEA1705914 | 25445 | Typhi |
| GCF_000245535.1 | ASM24553v1 | PRJNA80939 | SAMN02603101 | 2603101 | Typhi |
| GCF_000385905.1 | ASM38590v1 | PRJNA34855 | SAMN02603210 | 2603210 | Typhi |
| GCF_001048035.2 | ERL103914 | PRJEB3215 | SAMEA2072815 | 2363281 | Typhi |
| GCF_001048375.2 | M223 | PRJEB3215 | SAMEA2156512 | 2372886 | Typhi |
| GCF_001095585.2 | ERL114000 | PRJEB3215 | SAMEA2072817 | 2363290 | Typhi |
| GCF_001104165.2 | E00-7866 | PRJEB3215 | SAMEA2072798 | 2363309 | Typhi |
| GCF_001104885.2 | 10349_1#89_2 | PRJEB3215 | SAMEA2072799 | 2363310 | Typhi |
| GCF_001118185.2 | H12ESR00394-001A | PRJEB3215 | SAMEA2150110 | 2379332 | Typhi |
| GCF_001119245.2 | 76_1292 | PRJEB3215 | SAMEA1930246 | 2387556 | Typhi |
| GCF_001121865.2 | 404Ty | PRJEB3215 | SAMEA2072936 | 2363311 | Typhi |
| GCF_001127485.2 | ERL041834 | PRJEB3215 | SAMEA2072498 | 2363237 | Typhi |
| GCF_001135805.2 | ERL072973 | PRJEB3215 | SAMEA2072503 | 2363252 | Typhi |
| GCF_001148125.2 | ERL024120 | PRJEB3215 | SAMEA2072494 | 2363225 | Typhi |
| GCF_001148305.2 | ERL082356 | PRJEB3215 | SAMEA2072647 | 2363255 | Typhi |
| GCF_001163025.2 | H12ESR04734-001A | PRJEB3215 | SAMEA2072794 | 2363303 | Typhi |
| GCF_001165785.2 | ERL024919 | PRJEB3215 | SAMEA2072495 | 2363228 | Typhi |
| GCF_001302605.1 | ASM130260v1 | PRJNA286155 | SAMN03765654 | 3765654 | Typhi |
| GCF_001357935.2 | 80_2002 | PRJEB3215 | SAMEA1930249 | 2387559 | Typhi |
| GCF_001360555.2 | 2010_7898 | PRJEB3215 | SAMEA2058419 | 2301303 | Typhi |
| GCF_001362095.2 | 034151_4 | PRJEB3215 | SAMEA2072676 | 2363233 | Typhi |
| GCF_001362135.2 | 11909_3 | PRJEB3215 | SAMEA2072788 | 2363294 | Typhi |
| GCF_001362195.2 | H12ESR00755-001A | PRJEB3215 | SAMEA2072793 | 2363301 | Typhi |
| GCF_001362335.2 | Ty2 | PRJEB3215 | SAMEA2072934 | 2363305 | Typhi |
| GCF_003429465.1 | ASM342946v1 | PRJNA398278 | SAMN07507058 | 7507058 | Typhi |
| GCF_003716995.1 | ASM371699v1 | PRJNA474465 | SAMN09320528 | 9320528 | Typhi |
| GCF_003717015.1 | ASM371701v1 | PRJNA474465 | SAMN09320527 | 9320527 | Typhi |
| GCF_003717035.1 | ASM371703v1 | PRJNA474465 | SAMN09320526 | 9320526 | Typhi |
| GCF_003717055.1 | ASM371705v1 | PRJNA474465 | SAMN09320523 | 9320523 | Typhi |
| GCF_003717075.1 | ASM371707v1 | PRJNA474465 | SAMN09320522 | 9320522 | Typhi |
| GCF_003717095.1 | ASM371709v1 | PRJNA474465 | SAMN09320521 | 9320521 | Typhi |
| GCF_003717115.1 | ASM371711v1 | PRJNA474465 | SAMN09320520 | 9320520 | Typhi |
| GCF_003717135.1 | ASM371713v1 | PRJNA474465 | SAMN09320519 | 9320519 | Typhi |
| GCF_003717215.1 | ASM371721v1 | PRJNA474465 | SAMN09320518 | 9320518 | Typhi |
| GCF_003717285.1 | ASM371728v1 | PRJNA474465 | SAMN09320517 | 9320517 | Typhi |
| GCF_003717355.1 | ASM371735v1 | PRJNA474465 | SAMN09320516 | 9320516 | Typhi |
| GCF_003717395.1 | ASM371739v1 | PRJNA474465 | SAMN09320514 | 9320514 | Typhi |
| GCF_003717435.1 | ASM371743v1 | PRJNA474465 | SAMN09320513 | 9320513 | Typhi |
| GCF_003717455.1 | ASM371745v1 | PRJNA474465 | SAMN09320512 | 9320512 | Typhi |
| GCF_003717475.1 | ASM371747v1 | PRJNA474465 | SAMN09320511 | 9320511 | Typhi |
| GCF_003717515.1 | ASM371751v1 | PRJNA474465 | SAMN09320510 | 9320510 | Typhi |
| GCF_003717535.1 | ASM371753v1 | PRJNA474465 | SAMN09320509 | 9320509 | Typhi |
| GCF_003717575.1 | ASM371757v1 | PRJNA474465 | SAMN09320507 | 9320507 | Typhi |
| GCF_003717615.1 | ASM371761v1 | PRJNA474465 | SAMN09320564 | 9320564 | Typhi |
| GCF_003717635.1 | ASM371763v1 | PRJNA474465 | SAMN09320563 | 9320563 | Typhi |
| GCF_003717655.1 | ASM371765v1 | PRJNA474465 | SAMN09320562 | 9320562 | Typhi |
| GCF_003717675.1 | ASM371767v1 | PRJNA474465 | SAMN09320561 | 9320561 | Typhi |
| GCF_003717695.1 | ASM371769v1 | PRJNA474465 | SAMN09320560 | 9320560 | Typhi |
| GCF_003717715.1 | ASM371771v1 | PRJNA474465 | SAMN09320559 | 9320559 | Typhi |
| GCF_003717735.1 | ASM371773v1 | PRJNA474465 | SAMN09320558 | 9320558 | Typhi |
| GCF_003717755.1 | ASM371775v1 | PRJNA474465 | SAMN09320557 | 9320557 | Typhi |
| GCF_003717775.1 | ASM371777v1 | PRJNA474465 | SAMN09320556 | 9320556 | Typhi |
| GCF_003717795.1 | ASM371779v1 | PRJNA474465 | SAMN09320555 | 9320555 | Typhi |
| GCF_003717815.1 | ASM371781v1 | PRJNA474465 | SAMN09320554 | 9320554 | Typhi |
| GCF_003717835.1 | ASM371783v1 | PRJNA474465 | SAMN09320553 | 9320553 | Typhi |
| GCF_003717855.1 | ASM371785v1 | PRJNA474465 | SAMN09320552 | 9320552 | Typhi |
| GCF_003717875.1 | ASM371787v1 | PRJNA474465 | SAMN09320551 | 9320551 | Typhi |
| GCF_003717895.1 | ASM371789v1 | PRJNA474465 | SAMN09320550 | 9320550 | Typhi |
| GCF_003717915.1 | ASM371791v1 | PRJNA474465 | SAMN09320549 | 9320549 | Typhi |
| GCF_003717935.1 | ASM371793v1 | PRJNA474465 | SAMN09320548 | 9320548 | Typhi |
| GCF_003717955.1 | ASM371795v1 | PRJNA474465 | SAMN09320547 | 9320547 | Typhi |
| GCF_003717975.1 | ASM371797v1 | PRJNA474465 | SAMN09320546 | 9320546 | Typhi |
| GCF_003717995.1 | ASM371799v1 | PRJNA474465 | SAMN09320545 | 9320545 | Typhi |
| GCF_003718015.1 | ASM371801v1 | PRJNA474465 | SAMN09320544 | 9320544 | Typhi |
| GCF_003718035.1 | ASM371803v1 | PRJNA474465 | SAMN09320543 | 9320543 | Typhi |
| GCF_003718055.1 | ASM371805v1 | PRJNA474465 | SAMN09320542 | 9320542 | Typhi |
| GCF_003718075.1 | ASM371807v1 | PRJNA474465 | SAMN09320541 | 9320541 | Typhi |
| GCF_003718095.1 | ASM371809v1 | PRJNA474465 | SAMN09320540 | 9320540 | Typhi |
| GCF_003718115.1 | ASM371811v1 | PRJNA474465 | SAMN09320539 | 9320539 | Typhi |
| GCF_003718135.1 | ASM371813v1 | PRJNA474465 | SAMN09320538 | 9320538 | Typhi |
| GCF_003718155.1 | ASM371815v1 | PRJNA474465 | SAMN09320537 | 9320537 | Typhi |
| GCF_003718175.1 | ASM371817v1 | PRJNA474465 | SAMN09320536 | 9320536 | Typhi |
| GCF_003718195.1 | ASM371819v1 | PRJNA474465 | SAMN09320535 | 9320535 | Typhi |
| GCF_003718235.1 | ASM371823v1 | PRJNA474465 | SAMN09320533 | 9320533 | Typhi |
| GCF_003718255.1 | ASM371825v1 | PRJNA474465 | SAMN09320532 | 9320532 | Typhi |
| GCF_003718275.1 | ASM371827v1 | PRJNA474465 | SAMN09320531 | 9320531 | Typhi |
| GCF_003718295.1 | ASM371829v1 | PRJNA474465 | SAMN09320530 | 9320530 | Typhi |
| GCF_003718315.1 | ASM371831v1 | PRJNA474465 | SAMN09320529 | 9320529 | Typhi |
| GCF_003718355.1 | ASM371835v1 | PRJNA474465 | SAMN09320578 | 9320578 | Typhi |
| GCF_003718375.1 | ASM371837v1 | PRJNA474465 | SAMN09320577 | 9320577 | Typhi |
| GCF_003718395.1 | ASM371839v1 | PRJNA474465 | SAMN09320576 | 9320576 | Typhi |
| GCF_003718415.1 | ASM371841v1 | PRJNA474465 | SAMN09320575 | 9320575 | Typhi |
| GCF_003718435.1 | ASM371843v1 | PRJNA474465 | SAMN09320574 | 9320574 | Typhi |
| GCF_003718455.1 | ASM371845v1 | PRJNA474465 | SAMN09320573 | 9320573 | Typhi |
| GCF_003718475.1 | ASM371847v1 | PRJNA474465 | SAMN09320572 | 9320572 | Typhi |
| GCF_003718495.1 | ASM371849v1 | PRJNA474465 | SAMN09320571 | 9320571 | Typhi |
| GCF_003718515.1 | ASM371851v1 | PRJNA474465 | SAMN09320570 | 9320570 | Typhi |
| GCF_003718535.1 | ASM371853v1 | PRJNA474465 | SAMN09320569 | 9320569 | Typhi |
| GCF_003718555.1 | ASM371855v1 | PRJNA474465 | SAMN09320568 | 9320568 | Typhi |
| GCF_003718575.1 | ASM371857v1 | PRJNA474465 | SAMN09320567 | 9320567 | Typhi |
| GCF_003718595.1 | ASM371859v1 | PRJNA474465 | SAMN09320566 | 9320566 | Typhi |
| GCF_003718615.1 | ASM371861v1 | PRJNA474465 | SAMN09320565 | 9320565 | Typhi |
| GCF_003718635.1 | ASM371863v1 | PRJNA474465 | SAMN09320525 | 9320525 | Typhi |
| GCF_003718655.1 | ASM371865v1 | PRJNA474465 | SAMN09320506 | 9320506 | Typhi |
| GCF_003719215.1 | ASM371921v1 | PRJNA474465 | SAMN09320534 | 9320534 | Typhi |
| GCF_003719235.1 | ASM371923v1 | PRJNA474465 | SAMN09320508 | 9320508 | Typhi |
| GCF_003719255.1 | ASM371925v1 | PRJNA474465 | SAMN09320515 | 9320515 | Typhi |
| GCF_003719555.1 | ASM371955v1 | PRJNA471337 | SAMN09208111 | 9208111 | Typhi |
| GCF_004136335.1 | ASM413633v1 | PRJNA480202 | SAMN09630442 | 9630442 | Typhi |
| GCF_005885835.1 | ASM588583v1 | PRJNA543969 | SAMN11792777 | 11792777 | Typhi |
| GCF_900185485.1 | BL60006 | PRJEB21155 | SAMEA104109193 | 7190870 | Typhi |
| GCF_900205255.1 | 1554 | PRJEB5919 | SAMEA3109638 | 3338098 | Typhi |
| GCF_900205265.1 | lupe_GEN0059_5 | PRJEB5919 | SAMEA2564024 | 3071773 | Typhi |
| GCF_900205275.1 | 403Ty | PRJEB5919 | SAMEA2564027 | 3071775 | Typhi |
| GCF_900205295.1 | E98-3139 | PRJEB5919 | SAMEA2467787 | 3000319 | Typhi |
| GCF_901457615.1 | ERS3381924 | PRJEB32272 | SAMEA5577690 | 11516613 | Typhi |
