# Supplementary material for: The molecular basis of extensively drug-resistant Salmonella Typhi isolates from pediatric septicemia patients
Source: PLoS One. 2021 Sep 28;16(9):e0257744. doi: 10.1371/journal.pone.0257744 (PMC8478237; doi:10.1371/journal.pone.0257744)
Supplement: S6 Table — (DOCX) [file pone.0257744.s007.docx]

**S6 Table. Sequencing results associated with fluoroquinolone resistance, related to Fig 3.**

| Genes | Mutations | References | **Findings** |
| --- | --- | --- | --- |
| *gyrA* | M52L | [14] | **Wild type** |
|  | G81C | [16] | **Wild type** |
|  | D82G | [16] | **Wild type** |
|  | S83F/Y/L | [13, 20] | **S83F** |
|  | D87Y/H/N/G/A | [13, 20, 49] | **Wild type** |
|  | A119E | [16] | **Wild type** |
| *gyrB* | S464Y/F/T | [17] | **Wild type** |
|  | Q465L |  | **Wild type** |
|  | E466D |  | **Wild type** |
|  | A468E |  | **Wild type** |
| *parC* | T57S | [18, 19] | **Wild type** |
|  | G72S | [13] | **Wild type** |
|  | G78D | [16] | **Wild type** |
|  | D79G/R | [16, 18] | **Wild type** |
|  | S80R/I | [16, 20] | **Wild type** |
|  | G84G/K | [18, 20] | **Wild type** |
|  | W106G | [15] | **Wild type** |
| *parE* | D420N | [20] | **Wild type** |
|  | Y434S | [18] | **Wild type** |
|  | S458P | [16] | **Wild type** |

49. Baker S, Duy PT, Nga TV, Dung TT, Phat VV, Chau TT, et al. Fitness benefits in fluoroquinolone-resistant Salmonella Typhi in the absence of antimicrobial pressure. Elife. 2013;2:e01229.
