# Supplementary material for: The molecular basis of extensively drug-resistant Salmonella Typhi isolates from pediatric septicemia patients
Source: PLoS One. 2021 Sep 28;16(9):e0257744. doi: 10.1371/journal.pone.0257744 (PMC8478237; doi:10.1371/journal.pone.0257744)

The original uncropped images were obtained using an iBright CL1500 imager. Samples are PCR amplicons, as indicated in the method. Loading orders are directly displayed on the gels. L stands for the ladder. The same ladder was used for all gels. Which images were used for preparing which figures are indicated on the top of each gel image.

Ladder (L)

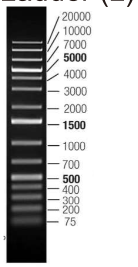

pltB gels 1-4 (Fig 1B)

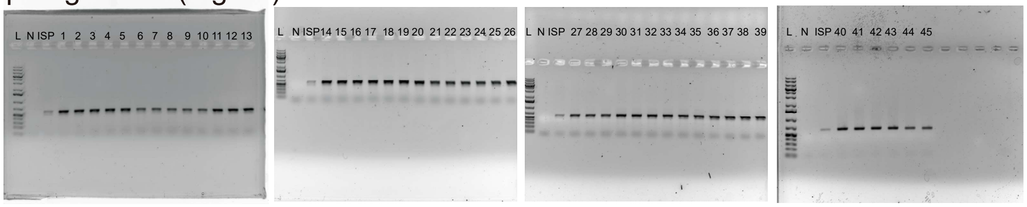

blaTEM1 gels 1-2 (Fig 2B)

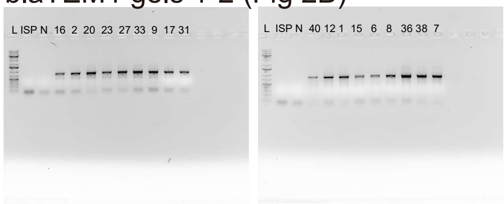

dhfR7 gels 1-2 (Fig 2C)

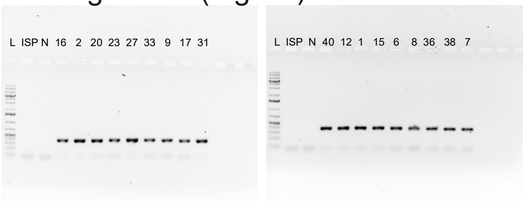

sul1 gels 1-2 (Fig 2C)

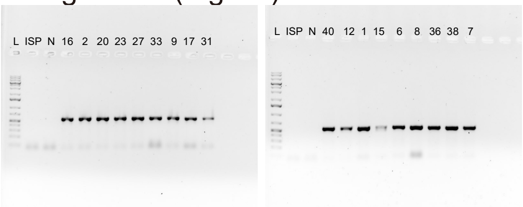

catA1 gels 1-2 (Fig 2D)

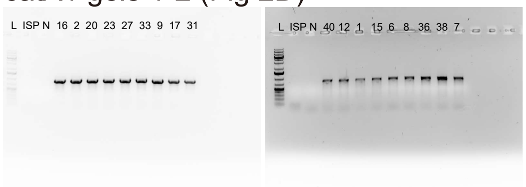

qnrS gels 1-2 (Fig 3A)

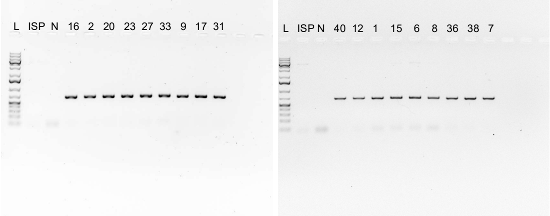

gyrA gels 1-2 (Fig 3B)

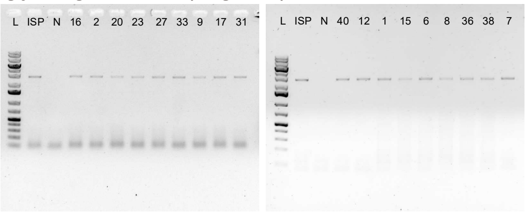

gyrB gels 1-2 (Fig 3C)

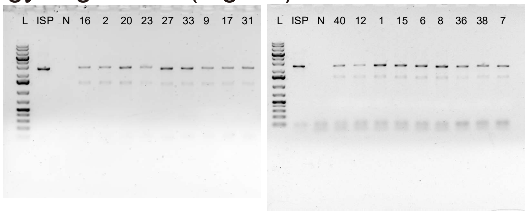

parC gels 1-2 (Fig 3D)

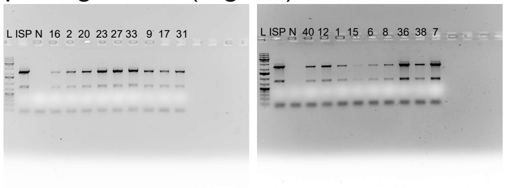

parE gels 1-2 (Fig 3E)

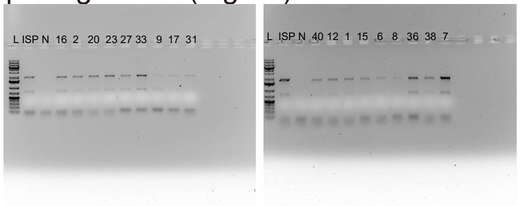

blaCTXM15 gels 1-2 (Fig 4)

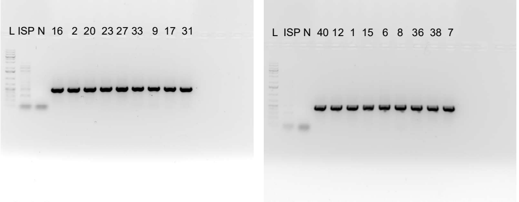

macA gels 1-2 (Fig 5A)

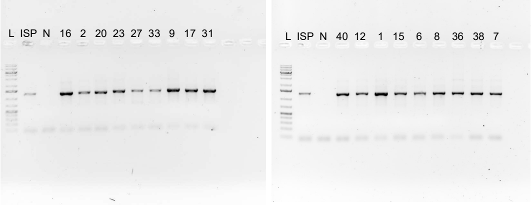

acrB-Nterm gels 1-2 (Fig 5B)

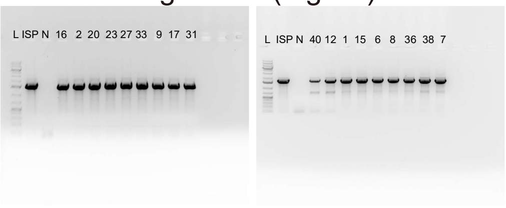

acrB-Cterm gels 1-2 (Fig 5B)

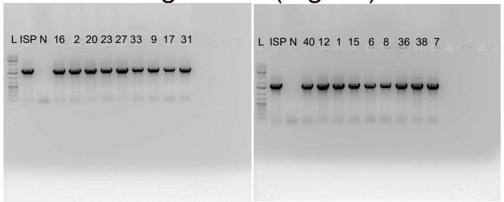

acrR gels 1-2 (Fig 6B)

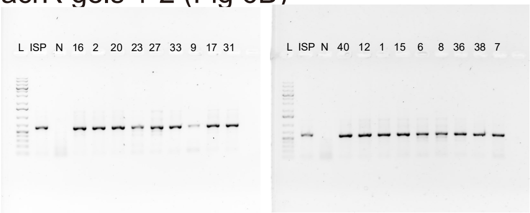

Supplement: S1 Raw images — (PDF) [file pone.0257744.s008.pdf]
